# Hyperplastic Gastric Polyp Causing Upper Gastrointestinal Hemorrhage and Acute Blood Loss Anemia

**DOI:** 10.7759/cureus.8494

**Published:** 2020-06-07

**Authors:** Aimen Farooq, Bayarmaa Mandzhieva, James Wert, Mamoon Ur Rashid

**Affiliations:** 1 Internal Medicine, AdventHealth Orlando, Orlando, USA

**Keywords:** hyperplastic polyp, acute blood loss anemia, endoscopy, upper gastrointestinal bleeding, hot snare polypectomy

## Abstract

Hyperplastic polyps are the second most common type of gastric polyp in the United States with equal incidence in both genders, usually found incidentally during endoscopic examinations. It is a well-known fact that they are associated with iron-deficiency anemia due to chronic blood loss. We present a case of a 69-year-old man with a relatively small hyperplastic gastric polyp with acute upper gastrointestinal bleeding, presenting with melena and acute blood loss anemia requiring admission to intensive care unit and urgent endoscopic intervention with hot snare removal of the polyp and cautery of surrounding area with excellent hemostasis. The pathology revealed focal intestinal metaplasia and low-grade dysplasia with no evidence of malignancy. He was subsequently discharged with outpatient follow-up with gastroenterology.

## Introduction

Hyperplastic polyps constitute 17% of all gastric polyps [1]. These polyps usually occur in the elderly with equal incidence in males and females [2]. They represent epithelial proliferations that primarily occur in the antrum of the stomach. On endoscopy, these polyps appear to be smooth and dome shaped. Larger lesions become lobulated or pedunculated with frequent erosions of surface epithelium [3]. The size may vary from one to twelve centimeters [4]. They are mostly asymptomatic, but larger hyperplastic polyps can cause symptoms, such as abdominal pain, chronic occult blood loss presenting as iron deficiency anemia or gastric outlet obstruction. Al-Haddad et al. showed that hyperplastic polyps of the gastric antrum are a rare but significant cause of gastrointestinal (GI) blood loss in older patients, and removal of the polyps using endoscopic or surgical methods may be required for resolution of the blood loss [5]. Gastric mucosal infection with Helicobacter pylori (H. pylori) has been reported in up to 90% of cases; however, our patient did not have any documented history of H. pylori [6].

Clinicians should be prepared for unusual presentations of polyps and be aware of this rare complication because time-sensitive endoscopic management can prevent bad outcomes.

## Case presentation

A 69-year-old male with past medical history of essential hypertension, diabetes mellitus type 2 and gastroesophageal reflux disease presented to the hospital with complaints of melena and diarrhea for the past two weeks. The patient noted that he has been having multiple episodes of watery, black stools. These episodes were associated with intermittent, generalized, crampy abdominal pain that resolved after each bowel movement. He also reported that his recent blood pressure (BP) has been running on the lower side in the 110s. He denied nausea, vomiting, fever, chills, travel, recent antibiotics or nonsteroidal anti-inflammatory drugs (NSAIDs) use.

The patient has been on long-term proton pump inhibitors (PPIs) for the past seven years and was not on any anticoagulation. Review of symptoms was otherwise negative except as mentioned.

**Figure 1:**
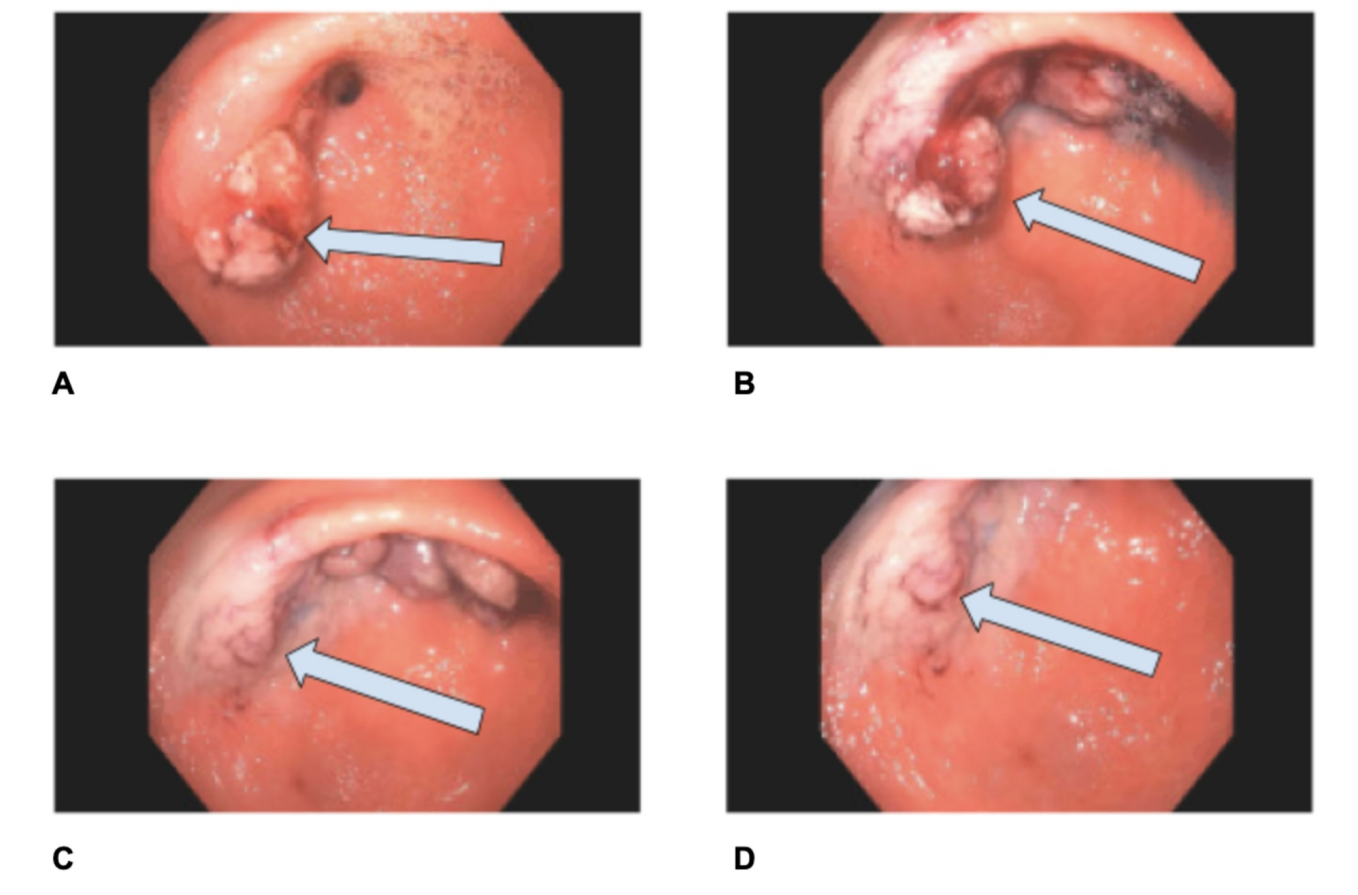
Initial hyperplastic polyp (A, B) A single greater than 50 millimeters sessile polyp with bleeding and stigmata of recent bleeding was found in the gastric antrum extending into the duodenal bulb from two different angles. (C, D) (different angles) The polyp was removed with a volume-indigocarmine-epinephrine 1:100,000 injection-lift technique using a hot snare.

Pertinent GI history was significant for a gastric polyp that was resected two months ago (Figure 1). Pathology report at that time showed a 50-millimeter ulcerated hyperplastic polyp without evidence of malignancy which was removed by hot snare.

Family history was positive for hypertension and heart disease on both maternal and paternal sides of the family, but no history of GI malignancies.

On initial evaluation in emergency department (ED), the patient was found to be afebrile, hypotensive with BP 87/41 mmHg, mean arterial pressure (MAP) 56 mmHg and heart rate 61 beats per minute. The patient was in mild distress and appeared diaphoretic, the cardiopulmonary exam was unremarkable and the abdomen was soft and nontender without organomegaly. Rectal exam revealed black stools, no signs of fissure, hemorrhoids or skin tags.

**Figure 2:**
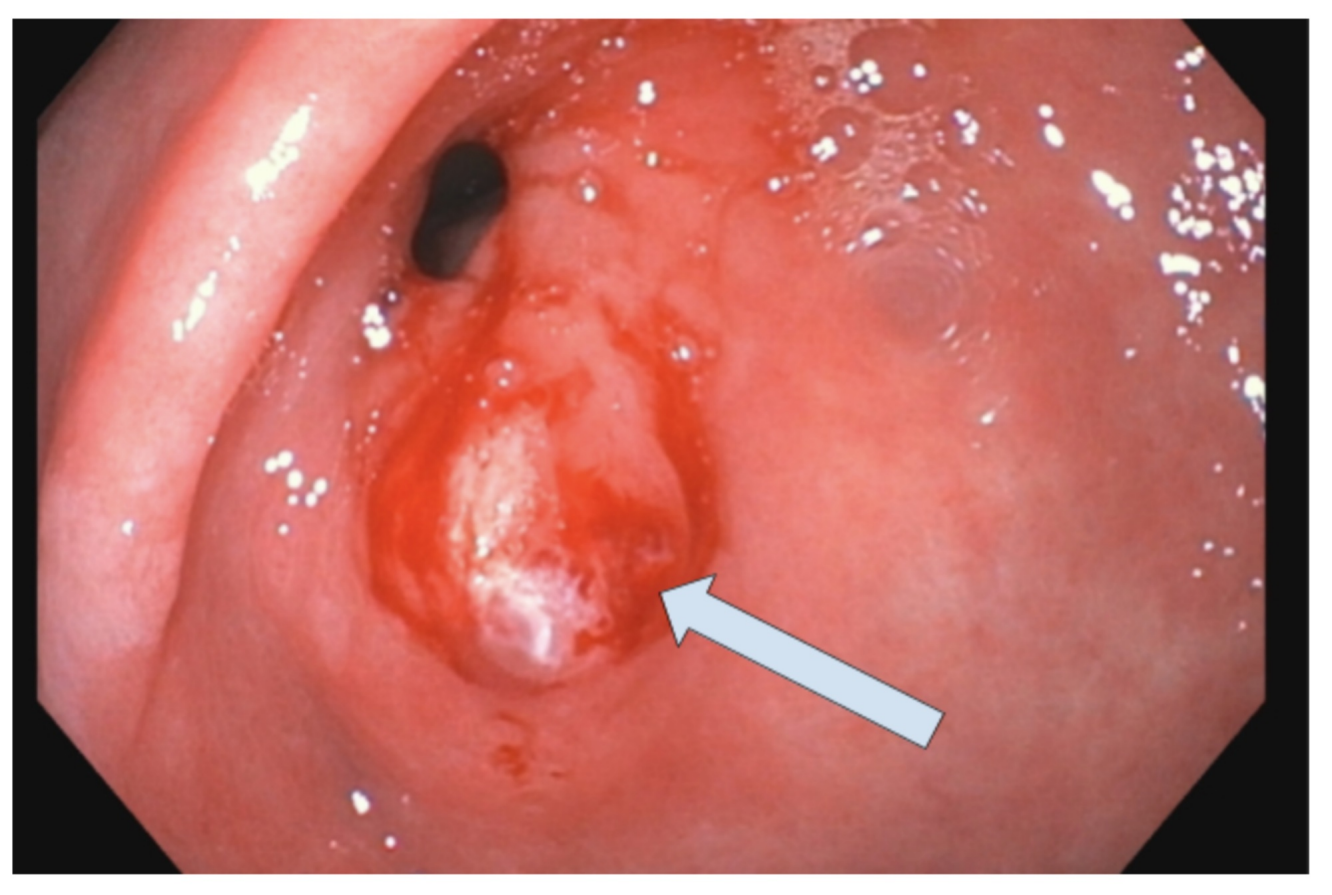
Residual gastric polyp A single six-millimeter sessile polyp with bleeding and stigmata of recent bleeding was found in the gastric antrum.

**Figure 3:**
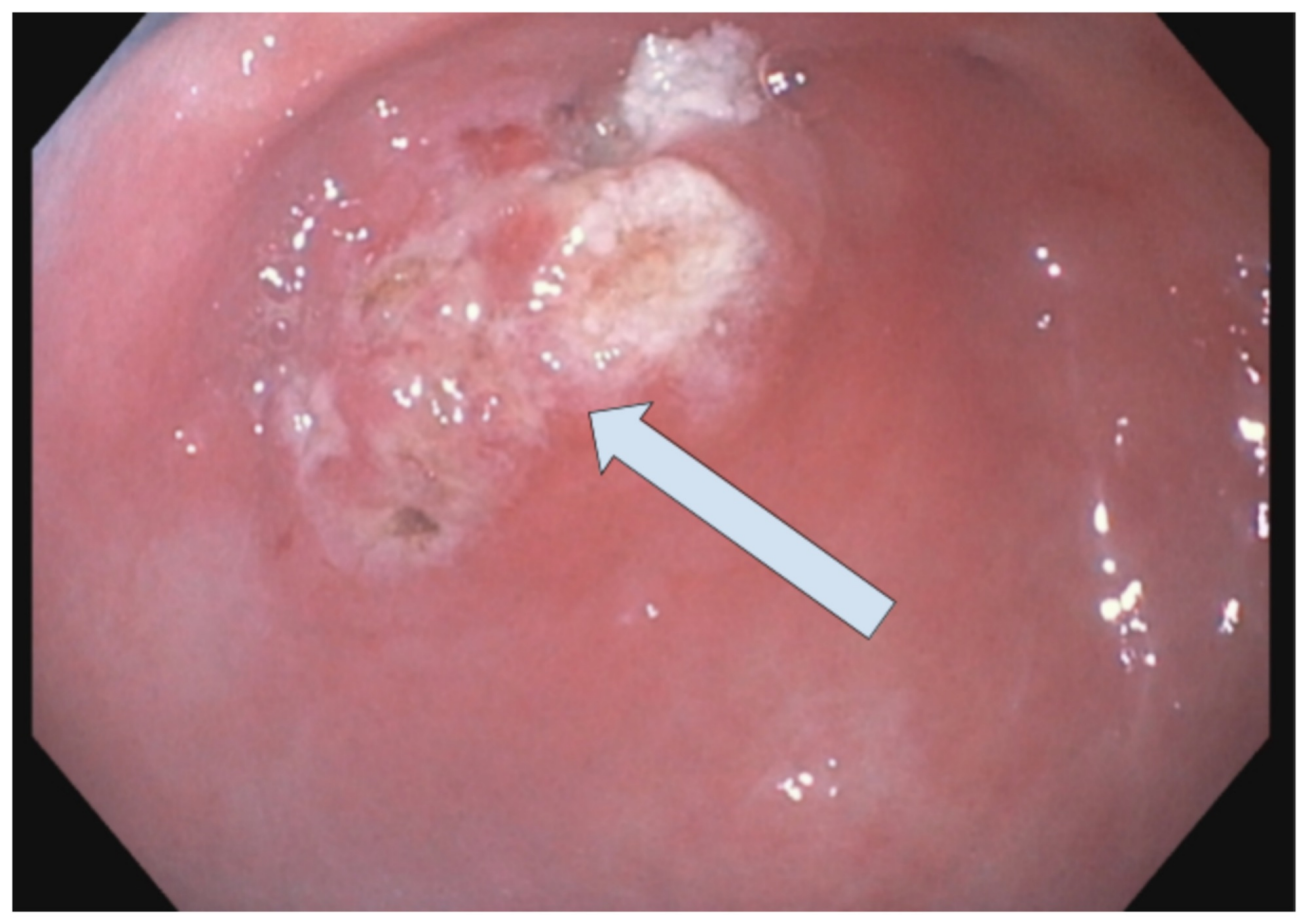
Polypectomy site The polyp was removed with a hot snare. Resection and retrieval were complete.

His initial labs revealed normocytic anemia with hemoglobin of 10.1 g/dl (normal range: 13.5-17.5 g/dl for men) and mean corpuscular volume (MCV) of 90.2 fl (normal range: 82-99 fl). There was evidence of acute kidney injury with creatinine of 1.97 mg/dl (normal range: 0.6-1.2 mg/dl) and blood urea nitrogen (BUN) of 32 mg/dl (normal range: 5-25 mg/dl). Chest X ray was done in the ED, which did not show evidence of acute cardiothoracic pathology. The patient received 1.5 L of normal saline bolus in the ED along with protonix 80 mg and was continued on protonix drip. BP increased to 110/46 mmHg with MAP 67 mmHg after fluid resuscitation, and the patient was then transferred to intensive care unit (ICU) for close monitoring, further workup and management of acute blood loss anemia and hypotension. Gastroenterology service was consulted from the ED, and the patient underwent emergent esophagoduodenoscopy that showed a six-millimeter residual, single gastric polyp with active bleeding (Figure 2), treated with hot snare and cautery of the surrounding area achieving excellent hemostasis (Figure 3).

**Figure 4:**
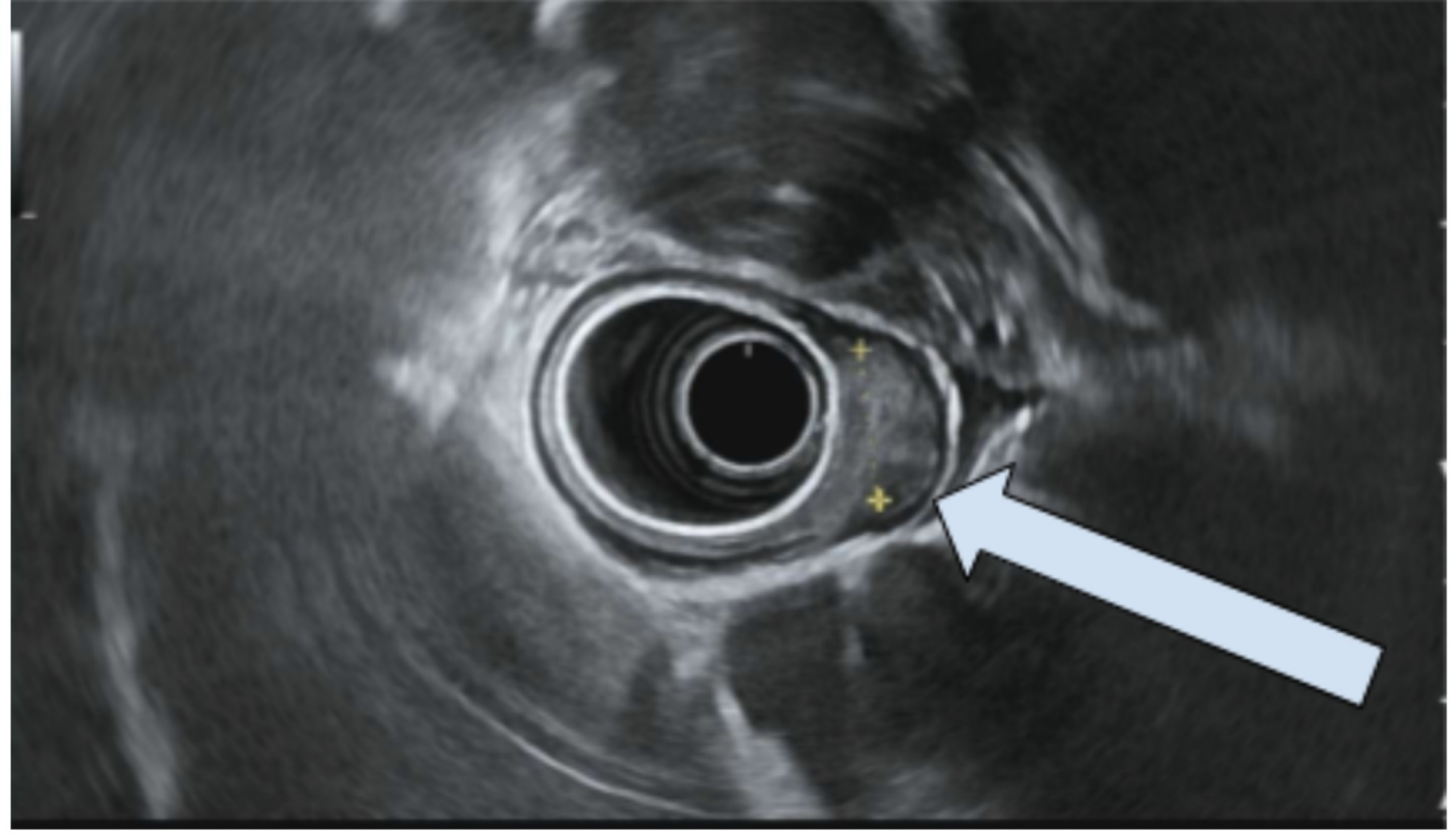
Upper endoscopic ultrasound Patchy wall thickening was visualized endosonographically in the antrum of the stomach. This appeared to primarily be due to thickening within the luminal interface/superficial mucosa (layer 1) and deep mucosa (layer 2). No intramural lesions were visualized underneath the mucosa.

There was a submucosal lesion present under the polyp site. Biopsies were taken and a surgical specimen was sent to pathology that revealed hyperplastic gastric polyp with intestinal metaplasia and low-grade dysplasia. No morphological and histochemical evidence of H. pylori infection was found. Repeat endoscopic ultrasound (EUS) was performed the next day to further delineate the submucosal lesion under the polyp site. EUS revealed patchy wall thickening in the antrum of the stomach, which appeared primarily due to thickening within the luminal interface/superficial mucosa (layer 1) and deep mucosa (layer 2). No intramural lesions were visualized underneath the mucosa (Figure 4).

The patient was transferred out of the ICU and did not have further episodes of bleeding or hypotension. He was discharged from the hospital in stable condition and advised to follow up with GI on an outpatient basis for a repeat surveillance esophagoduodenoscopy within the next three months. No further follow-up required for the submucosal lesion due to the lesion being small and asymptomatic. It was deemed that further evaluation will be required only if the patient becomes symptomatic or if there is an increase in the size of the lesion.

## Discussion

Hyperplastic polyps are the second most common type of gastric polyps in the United States following fundic gland polyps. They are usually asymptomatic and found incidentally during endoscopic examinations. The size of hyperplastic polyps ranges from a few millimeters to several centimeters. As the surfaces of large hyperplastic polyps can be eroded, they can cause iron-deficiency anemia due to chronic blood loss [1].

Hyperplastic gastric polyps are benign epithelial proliferations that often require no intervention. It has been suggested that regenerative responses significantly contribute to pathogenesis of hyperplastic polyps [7]. They are associated with H. pylori gastritis and atrophic autoimmune gastritis, which predispose the epithelium to chronic inflammation and epithelial repair [3].

These polyps can be solitary or numerous in number and are rarely associated with genetic conditions, such as familial juvenile polyposis, Peutz-Jeghers syndrome and Cronkhite-Canada syndrome [8]. They typically have dome-shaped morphology and smooth red surface during endoscopic evaluation. More prominent in size polyps can appear pedunculated or become lobulated and often develop erosions of the surface. Histologically, hyperplastic polyps consist of distorted, tortuous gastric foveolar epithelium set within an inflamed, edematous stroma [9].

It is very important for general physicians and gastroenterologists to remain aware of the fact that these polyps may be symptomatic or have neoplastic potential and be prepared for unusual presentations of polyps. Symptomatic hyperplastic polyps usually present with occult GI bleeding. Patients presenting with acute bleeding are rare especially outside the setting of anticoagulant therapy. Al-Haddad et al. demonstrated that 14 patients (1.4%) among 987 patients with suspected upper GI bleeding revealed hyperplastic polyps in the gastric antrum as the cause of bleeding, and five of the patients presented with melena [5]. Endoscopic evaluation and treatment should be immediately performed, which includes resection and eradication of H. pylori, when present.

We encountered a patient with a small hyperplastic polyp who presented with acute upper GI bleeding. This case is important because patients presenting with acute GI bleeding due to these polyps are very rare. Only a few cases that had similar clinical presentation are available in the literature. This case describes an adult male patient with one relatively small residual gastric polyp causing overt GI bleeding and melena, requiring ICU admission and emergent endoscopic intervention. As we stated above, he was not on antiplatelet or anticoagulant therapy. It is worth mentioning that our patient had a history of long-term PPI administration for his gastroesophageal reflux disease. In recent years, the increased risk of gastric polyps during long-term PPI administration has been a growing concern [10].

It is also very important to emphasize that it is crucial to investigate if such polyps have dysplastic or neoplastic foci. Goddard et al. reported that the rate of adenocarcinoma in hyperplastic polyps ranges from 0.6% to 2.1% [11].

Han et al. demonstrated that the size greater than one centimeter is a risk factor for malignant potential and these polyps should undergo endoscopic resection [12]. Kang et al. suggested that pedunculated appearance and synchronous dysplasia are also associated with the risk of neoplastic transformation [13]. Size exceeding two centimeters is defined as giant gastric polyp and as size increases, possibility of clinically significant signs and neoplastic transformation increases [14].

In our case, the polyp size was just six millimeters with no ulceration present and pathology was significant for low-grade dysplasia with no foci of adenocarcinoma. We chose hot snare polypectomy and cautery of the surrounding area with excellent hemostasis. Biopsy was negative for H. pylori infection, which is commonly associated with these polyps.

Available studies have shown that dysplastic changes can transform to adenocarcinoma, which makes an argument for a complete resection of such polyps [15]. Surgical removal may also be feasible in some cases, and iron supplementation is often needed. Careful follow-up endoscopy is required keeping in mind the possibility of recurrence and neoplastic transformation.

## Conclusions

Hyperplastic polyps of the gastric antrum are a rare but significant cause of GI blood loss and acute blood loss anemia in older patients. Additionally, it is necessary to investigate if such polyps have foci of dysplasia or adenocarcinoma. Treatment should be immediately planned during initial endoscopy in patients with emergencies to prevent short-term and long-term complications. Endoscopists should carefully inspect such polyps and consider complete wider resection.
